# Association of Use of GRADE, Protocol Registration, and Journal Impact Factor With Reporting and Methodological Quality of Systematic Reviews Published in Rehabilitation Journals: A Meta-Epidemiological Study

**DOI:** 10.7759/cureus.95695

**Published:** 2025-10-29

**Authors:** Takahiro Tsuge, Norio Yamamoto, Yosuke Tomita, Akikazu Hagiyama, Daijo Shiratsuchi, Masatsugu Okamura, Takao Kaneko, Kosuke Suzuki, Yuki Nakashima, Shunsuke Taito, Takashi Yorifuji

**Affiliations:** 1 Rehabilitation, Kurashiki Medical Center, Kurashiki, JPN; 2 Department of Epidemiology, Graduate School of Medicine, Dentistry and Pharmaceutical Sciences, Okayama University, Okayama, JPN; 3 Systematic Reviewers, Scientific Research WorkS Peer Support Group (SRWS-PSG), Osaka, JPN; 4 Orthopedic Surgery, Minato Medical Coop-Kyoritsu General Hospital, Nagoya, JPN; 5 Physical Therapy, Faculty of Health Care, Takasaki University of Health and Welfare, Takasaki, JPN; 6 Physical Medicine &amp; Rehabilitation, Okayama University Hospital, Okayama, JPN; 7 Physical Therapy, School of Health Sciences, Faculty of Medicine, Kagoshima University, Kagoshima, JPN; 8 Rehabilitation Medicine, School of Medicine, Yokohama City University, Yokohama, JPN; 9 Berlin Institute of Health Center for Regenerative Therapies (BCRT), Charité – Universitätsmedizin Berlin, Berlin, DEU; 10 Rehabilitation, Yamagata Prefectural Central Hospital, Yamagata, JPN; 11 Rehabilitation, Yamagata Saisei Hospital, Yamagata, JPN; 12 Rehabilitation Medicine, Hiroshima University Hospital, Hiroshima, JPN; 13 Rehabilitation, Hiroshima University Hospital, Hiroshima, JPN

**Keywords:** citation, grade, journal impact factor, methodological and reporting quality, prisma

## Abstract

This study aimed to identify factors associated with the reporting and methodological quality of systematic reviews (SRs) published in rehabilitation journals. We conducted a meta-epidemiological study as a secondary analysis of a previous study. The study protocol was registered in the Open Science Framework. We analyzed 219 SRs from rehabilitation journals published since 2020. We assessed reporting quality using the Preferred Reporting Items for Systematic reviews and Meta-Analysis (PRISMA) 2020 and methodological quality using A MeaSurement Tool to Assess systematic Reviews (AMSTAR) 2. Multiple linear regression and Spearman's correlation were used to identify factors associated with quality, including Grading of Recommendations Assessment, Development and Evaluation (GRADE) approach and the Journal Impact Factor (JIF). Multivariate analysis revealed PRISMA 2020 adherence was significantly associated with use of GRADE (β = 4.33; 95% confidence interval (CI): 3.24-5.42), protocol registration (β = 3.40; 95% CI: 2.32-4.47), and the JIF (2023) (β = 0.69; 95% CI: 0.42-0.95). AMSTAR 2 adherence was also significantly associated with use of GRADE (β = 2.52; 95% CI: 1.88-3.17), protocol registration (β = 2.07; 95% CI: 1.44-2.70), and the JIF (2023) (β = 0.29; 95% CI: 0.14-0.45). Weak positive correlations were observed between the JIF (2023) and both PRISMA 2020 and AMSTAR 2 adherence (ρ = 0.27 and ρ = 0.22, respectively; both P < 0.01). It should be noted that these findings reflect associations and do not imply causality. To enhance the quality of SRs in rehabilitation, researchers should prioritize adherence to PRISMA 2020, particularly the use of GRADE and protocol registration, which this study identified as key associated factors.

## Introduction and background

The importance of systematic reviews (SRs) in medical decision-making, research planning, and clinical policy development continues to grow, as they are valued for their ability to synthesize extensive research findings [1,2]. To ensure that recommendations and decisions derived from SRs are less prone to bias, both complete reporting and robust methodology are essential [3,4]. The Preferred Reporting Items for Systematic reviews and Meta-Analysis (PRISMA) 2020 statement is the primary guideline for assessing reporting quality, while the A MeaSurement Tool to Assess systematic Reviews (AMSTAR) is a common tool for evaluating methodological quality [5,6].

Several factors may influence the reporting and methodological quality of SRs, including journal endorsement of PRISMA 2020, use of Grading of Recommendations Assessment, Development and Evaluation (GRADE), protocol registration, and the Journal Impact Factor (JIF). Previous studies have reported that application of PRISMA 2020 has led to improvements in the quality of SRs, suggesting it is a key influential factor [7,8]. The JIF is widely used to evaluate the quality of scientific journals and individual publications [9,10]. Prior studies suggest a positive association between JIF and the methodological quality of SRs in core clinical journals [11]. Nevertheless, this association is not universally observed. For instance, no such correlation has been found for SRs in the field of low back pain [12]. The specific relationship between JIF and methodological quality in rehabilitation journals is not well understood, as these associations can differ by field. Additionally, although an association between JIF and PRISMA endorsement has been noted, the relationship between JIF and adherence to PRISMA 2020 has not been explored [12].

Therefore, the aim of this study was to investigate factors associated with the reporting and methodological quality of SRs published in rehabilitation journals, including adherence to PRISMA 2020, use of GRADE, protocol registration, and the JIF.

## Review

Methods

Study Design and Protocol

We conducted a secondary analysis of a previous study, employing a meta-epidemiological study design [7]. This report adheres to the guidelines for meta-epidemiological studies (Table 1) [13]. The protocol for this study was prospectively registered at Open Science Framework (https://osf.io/f6wj9). As this study exclusively utilized publicly available data, neither ethical approval nor patient consent was required.

**Table 1:** Items for reporting methodological research, adapted from the PRISMA checklist PRISMA: Preferred Reporting Items for Systematic reviews and Meta-Analyses

Section/topic	#	Checklist item	Reported on page #
TITLE			
Title	1	Identify the report as a meta-epidemiologic study.	1
ABSTRACT			
Structured summary	2	Provide a structured summary that includes the background of the topic, goal of the study, data sources, method of data selection, appraisal and synthesis methods, results, limitations, conclusions and implications of key findings.	1
INTRODUCTION			
Rationale	3	Describe the rationale for the meta-epidemiological study in the context of what is already known.	1, 2
Objectives	4	Provide an explicit statement of the goal of the meta-epidemiological study and the hypothesis being empirically tested.	2
METHODS			
Protocol	5	Indicate if a protocol exists, if and where it can be accessed (eg, web address). Registration of a protocol is not mandatory.	2
Eligibility criteria	6	Specify study characteristics used as criteria for eligibility with a rationale.	3
Information sources	7	Describe all information sources (eg, databases with dates of coverage, contact with experts to identify additional studies, Internet searches) and search date.	3
Search	8	Present full electronic search strategy for at least one database, including any limits used, such that it could be repeated. Search is commonly not driven by a clinical question.	3
Study selection	9	Describe the process for selecting studies for inclusion (ie, how many reviewers selected studies, reviewing in duplicate or by single individuals).	3
Data collection process	10	Describe method of data extraction from reports (eg, piloted forms, independently, in duplicate) and any processes used for manipulating data or obtaining and confirming data from investigators.	4
Data items	11	List and define all variables for which data were sought and any assumptions and imputations made.	4
Risk of bias in individual studies	12	If risk of bias assessment of individual studies was relevant to the analysis, describe the items used and how this information is to be used during data synthesis.	NA
Summary measures	13	State the principal summary measures (eg, ratio of risk ratios, difference in means) and explain its meaning and direction to readers.	4, 5
Synthesis of results	14	Describe the statistical or descriptive methods of synthesis including measures of consistency if relevant. If applicable, describe the development of statistical or simulation modelling based on theoretical background. Describe and justify assumptions and computational approximations. Describe methods of additional analyses (eg, sensitivity or subgroup analyses, meta-regression), if done, indicating which were prespecified.	4, 5
RESULTS			
Study selection	15	Give numbers of studies assessed for eligibility and included in the study, with reasons for exclusions at each stage, ideally with a flow diagram. Present a measure of inter-reviewer agreement (eg, kappa statistic).	5, Figure 1
Study characteristics	16	For each study, present characteristics for which data were extracted and provide the citations. Clinical characteristics may not always be relevant.	5 Table 2, Table 3
Risk of bias within studies	17	If risk of bias assessment of individual studies was used in the meta-epidemiological analysis, report risk of bias indicators of each study to allow replication of findings.	NA
Results of individual studies	18	Present data elements used in the meta-epidemiological analysis from each study (results of clinical outcomes may not be relevant).	NA
Synthesis of results	19	Present results of statistical analysis done, including measures of precision and measures of consistency. Present validity of assumptions and fit of statistical or simulation modelling, if applicable.	8, 9, Table 4,5 Figure 2-8
Additional analysis	20	Give results of additional analyses, if done (eg, sensitivity or subgroup analyses, meta-regression).	NA
DISCUSSION			
Summary of evidence	21	Summarize the main findings and compare them with existing knowledge about the topic. The quality of evidence may not be relevant; however, investigators should describe their certainty in the results to readers.	14
Limitations	22	Discuss limitations at research methodology level (eg, likelihood of reporting or publication bias).	14
Conclusions	23	Provide general interpretation of the results and implications for future research. Provide any plausible impact on clinical practice.	14
FUNDING			
Funding	24	Describe sources of funding for the methodology research and role of funders.	15

Study Selection and Screening

This study was a secondary analysis of a dataset from a previous meta-epidemiological study [7]. The original search for this dataset was conducted using the MEDLINE (PubMed) database, which identified 240 SRs from 150 rehabilitation-specific journals published since 2020. For inclusion in the original dataset, SRs had to be published in English and include a pairwise meta-analysis on the effects of a health intervention. Studies such as individual patient data analyses, network meta-analyses, and scoping reviews were excluded. 

**Figure 1:**
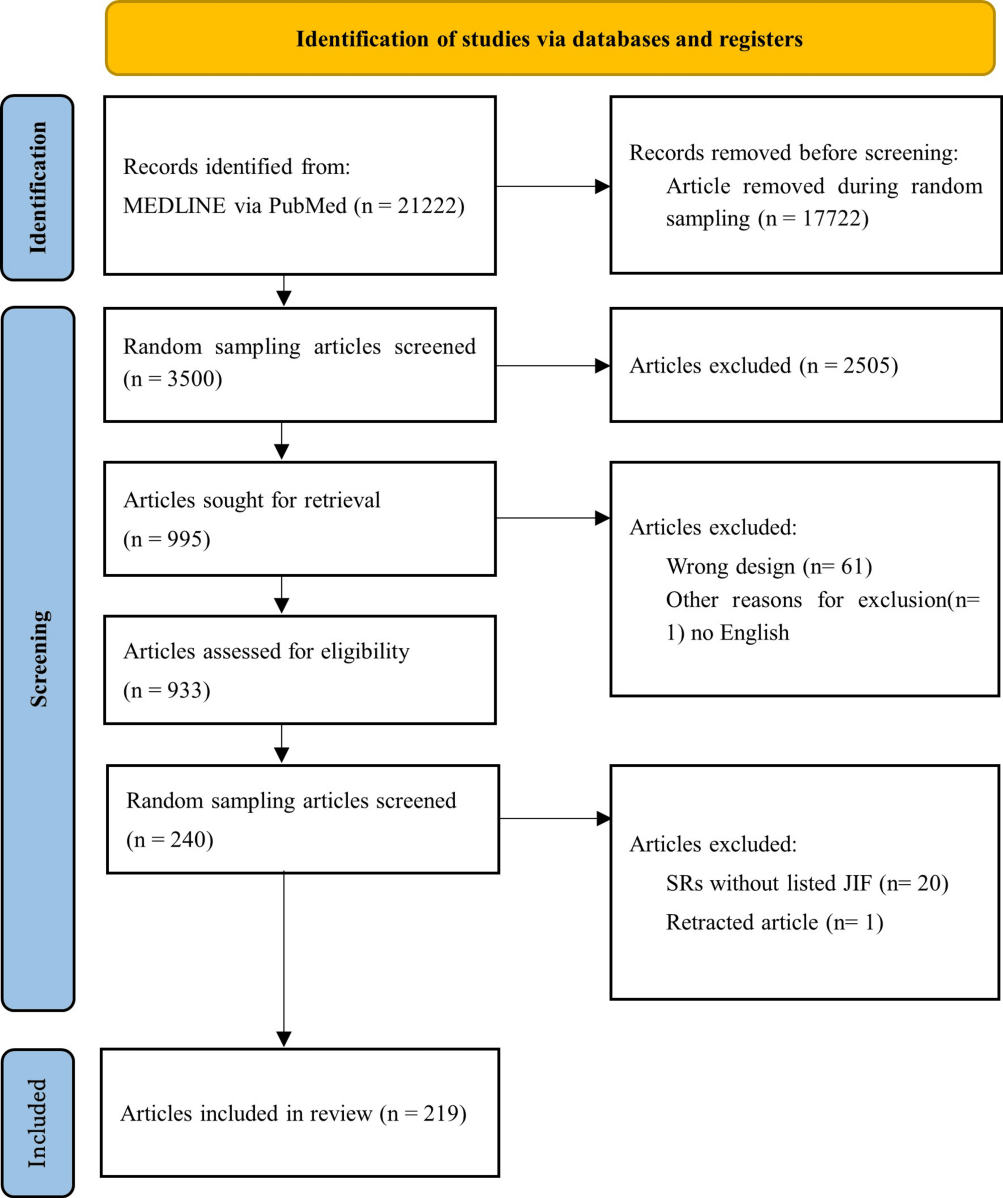
PRISMA 2020 flow diagram PRISMA: Preferred Reporting Items for Systematic reviews and Meta-Analyses

For the purpose of the present study, we applied further exclusion criteria to this initial dataset. We excluded 20 SRs that did not have a JIF listed in 2023 and one SR that had been retracted. Consequently, a total of 219 SRs were included in the final analysis (Figure 1).

Data Extraction

Relevant information was extracted from each included SR independently by two of the eight reviewers (T.T., Y.T., A.H., D.S., M.O., T.K., K.S., and Y.N.) using a standardized data collection form. Prior to the full data extraction, a calibration exercise was conducted where the reviewers pilot-tested five randomly selected articles to ensure a consistent understanding of the evaluation criteria. The following data points were collected: publication years, the geographic region of the first author's affiliated institution, number of authors, number of included studies, protocol registration, funding support, reported conflicts of interest, reported use of PRISMA 2020, PRISMA endorsement within the journal’s "Instructions to Authors" or on its website, and use of GRADE [12,14]. Furthermore, the JIF (2023) and citation density (calculated as the average number of citations per year) were extracted [15,16]. Reporting quality was evaluated based on adherence to PRISMA 2020, while methodological quality was assessed using adherence to AMSTAR 2 [7]. In that process, two independent reviewers assessed the articles. The overall adherence score for PRISMA 2020 was operationalized as the total number of 'Yes' ratings. For AMSTAR 2, both 'Yes' and 'Partial Yes' ratings were counted as 'Yes' for the total score. Any disagreements encountered during the data extraction and quality assessment process were resolved through discussion. If a consensus could not be reached, a third reviewer (N.Y. or S.T.) was consulted for a final decision.

Data Analysis

Categorical data were summarized as frequencies and continuous data as medians and interquartile ranges (IQR) or as means and SDs, depending on data distribution. The possible relationships between adherence to PRISMA 2020 or AMSTAR 2 as dependent variables and the following independent variables were examined: geographic region of the first author's institution, number of authors, number of included studies, protocol registration, funding support, conflicts of interest, reported use of PRISMA 2020, PRISMA endorsement, use of GRADE, citation density, and JIF (2023). These relationships were analyzed using simple and multiple linear regression models, with adjustment for other independent variables in the latter. Spearman's rank correlation coefficients were calculated for the following pairs: JIF and PRISMA 2020 adherence, JIF and AMSTAR 2 adherence, citation density and PRISMA 2020 adherence, and citation density and AMSTAR 2 adherence. The correlation coefficient values were interpreted according to the following criteria: <0.20 as very weak, 0.20-0.39 as weak, 0.40-0.59 as moderate, 0.60-0.79 as strong, and ≥0.80 as very strong [17,18]. We employed both multiple linear regression and Spearman's correlation analyses to provide a comprehensive assessment of factors associated with SRs quality. Multiple linear regression was used to identify independent predictors of quality scores while adjusting for potential confounding variables. In contrast, Spearman's correlation was used to specifically evaluate the simple, unadjusted strength and direction of the relationship between quality scores and key continuous variables like JIF and citation density. To check for multicollinearity among the independent variables in our regression models, we calculated the Variance Inflation Factor (VIF). In all final models, the VIF for each variable was well below the common threshold of 5 (the maximum observed VIF was 1.57), indicating that multicollinearity was not a significant concern [19]. Two-tailed p-values of less than 0.05 were considered statistically significant. All analyses were performed using Stata, ver. 17.0 (StataCorp LLC, USA).

Results

Characteristics of the SRs included in this study are summarized in Tables 2 and 3. Asia was the most common geographical region of the first author's institution (n = 81, 37.0%), followed by Europe (n = 70, 32.0%), and Central and South America (n = 27, 12.3%). Among the included SRs, 146 (66.7%) had a registered protocol, 107 (48.9%) reported use of PRISMA 2020, 79 (36.1%) use of GRADE, and 156 (71.2%) were in journals that indicated PRISMA endorsement within their ‘Instructions to Authors’ or on their website. The median citation density was 3.0 (IQR 1.7-5.3), while the median JIF (2023) was 2.8 (IQR 2.2-3.6).

**Table 2:** Characteristics of included SRs SR: Systematic review; PRISMA: Preferred Reporting Items for Systematic reviews and Meta-Analyses; GRADE: Grading of Recommendations Assessment, Development and Evaluation; IQR: Interquartile range; JIF: Journal Impact Factor

Characteristic	Category	n	(%)
Publication years	2020	33	15.1
	2021	36	16.4
	2022	101	46.1
	2023	29	13.2
	2024	20	9.1
Geographic region of the first author's institution	Asia	81	37.0
	Europe	70	32.0
	Central and South America	27	12.3
	North America	21	9.6
	Oceania	10	4.6
	Middle East	9	4.1
	Africa	1	0.5
Number of authors	< 5	56	25.6
	≥ 5	163	74.4
Number of included studies	< 10	64	29.2
	≥ 10	155	70.8
Protocol registration	Yes	146	66.7
	No	73	33.3
Funding support	Yes	106	48.4
	No	74	33.8
	No information	39	17.8
Conflict of interest	Yes	20	9.1
	No	187	85.4
	No information	12	5.5
Reported use of PRISMA 2020	Yes	107	48.9
	No	112	51.1
PRISMA endorsement	Yes	156	71.2
	No	63	28.8
Use of GRADE	Yes	79	36.1
	No	140	63.9
Citation density	Median (IQR)	3.0	(1.7, 5.3)
JIF (2023)	Median (IQR)	2.8	(2.2, 3.6)

**Table 3:** Adherence to PRISMA 2020, PRISMA for abstracts, and AMSTAR 2, stratified by characteristics of included systematic reviews PRISMA: Preferred Reporting Items for Systematic reviews and Meta-Analyses; AMSTAR: A MeaSurement Tool to Assess systematic Reviews; IQR: Interquartile range; GRADE: Grading of Recommendations Assessment, Development and Evaluation

Characteristic	Category	PRISMA 2020 adherence		PRISMA 2020 for abstracts adherence		AMSTAR 2 adherence	
		Median	IQR	Median	IQR	Median	IQR
Geographic region of the first author's institution	Asia	28.0	25.0, 32.0	5.0	4.0, 6.0	10.0	8.0, 12.0
	Europe	28.0	25.0, 33.0	5.0	4.0, 6.0	10.0	8.0, 12.0
	Central and South America	24.0	20.0, 28.0	5.0	4.0, 6.0	9.0	7.0, 11.0
	North America	26.0	23.0, 28.0	5.0	4.0, 6.0	9.0	7.0, 13.0
	Oceania	28.0	25.0, 32.0	6.0	5.0, 6.0	9.5	8.0, 12.0
	Middle East	29.0	28.0, 34.0	5.0	5.0, 6.0	10.0	10.0, 15.0
	Africa	26.0	26.0, 26.0	4.0	4.0, 4.0	7.0	7.0, 7.0
Number of authors	< 5	26.0	24.5, 28.5	5.0	4.0, 5.5	9.0	7.0, 10.0
	≥ 5	28.0	25.0, 33.0	5.0	4.0, 6.0	10.0	8.0, 13.0
Number of included studies	< 10	25.5	23.0, 28.5	5.0	4.0, 6.0	9.0	7.0, 11.0
	≥ 10	28.0	26.0, 33.0	5.0	4.0, 6.0	10.0	8.0, 12.0
Protocol registration	Yes	29.0	26.0, 33.0	5.0	5.0, 6.0	11.0	9.0, 13.0
	No	25.0	21.0, 27.0	4.0	4.0, 5.0	8.0	6.0, 9.0
Funding support	Yes	29.0	25.0, 31.0	4.0	4.0, 6.0	10.0	8.0, 12.0
	No	28.0	25.0, 31.0	4.0	4.0, 6.0	10.0	8.0, 12.0
	No information	25.0	21.0, 28.0	5.0	4.0, 5.0	8.0	7.0, 11.0
Conflict of interest	Yes	27.5	26.0, 30.0	5.0	4.0, 6.0	9.5	7.5, 11.5
	No	28.0	24.0, 32.0	5.0	4.0, 6.0	10.0	8.0, 12.0
	No information	27.0	25.0, 32.0	5.0	4.5, 6.0	9.5	7.5, 11.5
Reported use of PRISMA 2020	Yes	28.0	26.0, 32.0	5.0	4.0, 6.0	10.0	8.0, 12.0
	No	27.0	24.0, 31.5	5.0	4.0, 6.0	10.0	7.5, 12.0
PRISMA endorsement	Yes	28.0	25.0, 32.0	5.0	4.0, 6.0	10.0	8.0, 12.0
	No	28.0	23.0, 30.0	5.0	4.0, 5.0	9.0	7.0, 11.0
Use of GRADE	Yes	32.0	28.0, 35.0	6.0	5.0, 7.0	12.0	10.0, 14.0
	No	26.0	23.0, 29.0	5.0	4.0, 5.0	8.0	7.0, 10.5

Table 4 present the results of univariate and multivariate linear regression analyses examining the association between characteristics of SRs and PRISMA 2020 adherence, PRISMA 2020 for abstracts adherence, and AMSTAR 2 adherence. The results of the multivariate analysis indicated significant associations for all three adherence. Specifically, PRISMA 2020 adherence was significantly associated with use of GRADE (β = 4.33; 95% confidence interval (CI): 3.24-5.42), protocol registration (β = 3.40; 95% CI: 2.32-4.47), and the JIF (2023) (β = 0.69; 95% CI: 0.42-0.95). PRISMA 2020 for abstracts adherence showed a significant association with protocol registration (β = 0.78; 95% CI: 0.40-1.17) and use of GRADE (β = 0.64; 95% CI: 0.24-1.03). AMSTAR 2 adherence was significantly associated with use of GRADE (β = 2.52; 95% CI: 1.88-3.17), protocol registration (β = 2.07; 95% CI: 1.44-2.70), and the JIF (2023) (β = 0.29; 95% CI: 0.14-0.45). In contrast, reported use of PRISMA 2020, PRISMA endorsement, and citation density were not significantly associated with any of the adherence.

**Table 4:** Univariate and multivariable linear regression analysis examining the association between SR characteristics and PRISMA 2020, PRISMA 2020 for abstracts, and AMSTAR 2 adherence * P < 0.05; ** P < 0.01; † Adjusted for geographic region of the first author's institution, number of authors, number of included studies, protocol registration, funding support, conflict of interest, reported use of PRISMA 2020, PRISMA endorsement, use of GRADE, citation density, JIF (2023) (excluding the variable being examined). SR: Systematic review; PRISMA: Preferred Reporting Items for Systematic reviews and Meta-Analyses; AMSTAR: A MeaSurement Tool to Assess systematic Reviews; GRADE: Grading of Recommendations Assessment, Development and Evaluation; JIF: Journal Impact Factor

Characteristic	Category	PRISMA 2020 adherence				PRISMA 2020 for abstracts adherence				AMSTAR 2 adherence			
		Univariate		Multivariate †		Univariate		Multivariate †		Univariate		Multivariate †	
		β	95% CI	β	95% CI	β	95% CI	β	95% CI	β	95% CI	β	95% CI
Protocol registration	Yes	4.88 **	3.59, 6.17	3.40 **	2.32, 4.47	0.99 **	0.60, 1.37	0.78 **	0.40, 1.17	2.85 **	2.13, 3.57	2.07 **	1.44, 2.70
	No	Ref.		Ref.		Ref.		Ref.		Ref.		Ref.	
Reported use of PRISMA 2020	Yes	1.30	-0.06, 2.65	0.45	-0.54, 1.45	0.18	-0.20, 0.56	0.11	-0.25, 0.47	0.38	-0.39, 1.14	-0.07	-0.65, 0.51
	No	Ref.		Ref.		Ref.		Ref.		Ref.		Ref.	
PRISMA endorsement	Yes	1.31	-0.19, 2.80	-0.06	-1.17, 1.05	0.63	0.22, 1.04	0.46	0.06, 0.86	0.92 *	0.08, 1.75	0.25	-0.40, 0.90
	No	Ref.		Ref.		Ref.		Ref.		Ref.		Ref.	
Use of GRADE	Yes	5.37 **	4.15, 6.60	4.33 **	3.24, 5.42	0.86 **	0.49, 1.24	0.64 **	0.24, 1.03	3.23 **	2.56, 3.90	2.52 **	1.88, 3.17
	No	Ref.		Ref.		Ref.		Ref.		Ref.		Ref.	
Citation density		0.13	-0.030, 0.29	0.0095	-0.12, 0.13	0.041	-0.0027, 0.086	0.032	-0.013, 0.077	-0.0090	-0.099, 0.081	-0.054	-0.13, 0.019
JIF (2023)		0.93 **	0.62, 1.24	0.69 **	0.42, 0.95	0.15 **	0.06, 0.24	0.09	-0.0091, 0.18	0.41 **	0.23, 0.587	0.29 **	0.14, 0.45
Geographic region of the first author's institution	Asia	-0.84	-2.42, 0.76	1.32 *	0.12, 2.51	-0.15	-0.61, 0.31	0.33	-0.12, 0.78	-0.34	-1.26, 0.58	0.66	-0.06, 1.38
	Europe	Ref.		Ref.		Ref.		Ref.		Ref.		Ref.	
	Central and South America	-4.29 **	-6.50, -2.08	-2.71 **	-4.34, -1.07	-0.20	-0.84, 0.43	0.14	-0.48, 0.75	-0.92	-2.20, 0.36	-0.50	-1.49, 0.48
	North America	-2.79 *	-5.21, -0.36	-1.51	-3.33, 0.31	-0.13	-0.83, 0.57	0.29	-0.40, 0.97	-0.81	-2.21, 0.59	-0.13	-1.23, 0.97
	Middle East	1.60	-1.86, 5.05	1.77	-0.68, 4.22	0.32	-0.68, 1.31	0.44	-0.48, 1.37	1.19	-0.81, 3.19	1.09	-0.39, 2.56
	Oceania	-0.77	-4.07, 2.52	-2.31	-4.67, 0.05	0.57	-0.38, 1.52	0.32	-0.57, 1.21	-0.34	-2.25, 1.57	-1.24	-2.66, 0.18
	Africa	-3.07	-12.9, 6.76	2.56	-4.45, 9.57	-1.12	-3.96, 1.71	-0.19	-2.83, 2.45	-3.14	-8.83, 2.54	0.20	-4.03, 4.43
Conflict of interest	Yes	-0.12	-2.50, 2.26	-0.67	-2.40, 1.06	-0.12	-0.78, 0.54	-0.22	-0.85, 0.40	0.03	-1.30, 1.36	-0.14	-1.16, 0.87
	No	Ref.		Ref.		Ref.		Ref.		Ref.		Ref.	
	No information	-0.43	-3.45, 2.58	-0.75	-2.99, 1.49	0.18	-0.66, 1.02	-0.17	-0.98, 0.64	-0.45	-2.14, 1.24	-1.32 *	-2.63, -0.0038
Number of authors	< 5	Ref.		Ref.		Ref.		Ref.		Ref.		Ref.	
	≥ 5	1.77 *	0.23, 3.32	-0.11	-1.25, 1.04	0.09	-.343, 0.53	-0.20	-0.62, 0.21	1.54 **	0.69, 2.39	0.49	-0.18, 1.16
Number of included studies	< 10	Ref.		Ref.		Ref.		Ref.		Ref.		Ref.	
	≥ 10	3.11 **	1.67, 4.55	1.72 **	0.64, 2.81	0.12	-0.30, 0.54	-0.14	-0.54, 0.25	0.93 **	0.10, 1.76	0.34	-0.30, 0.98
Funding support	Yes	0.84	-0.61, 2.30	0.24	-0.88, 1.36	0.22	-0.20, 0.64	0.34	-.073, 0.75	0.32	-0.52, 1.16	0.20	-0.47, 0.87
	No	Ref.		Ref.		Ref.		Ref.		Ref.		Ref.	
	No information	-3.53 **	-5.44, -1.63	-2.24 **	-3.73, -0.76	-0.30	-0.85, 0.26	0.01	-0.53, 0.55	-1.28 *	-2.38, -0.18	-0.44	-1.32, 0.45

**Figure 2:**
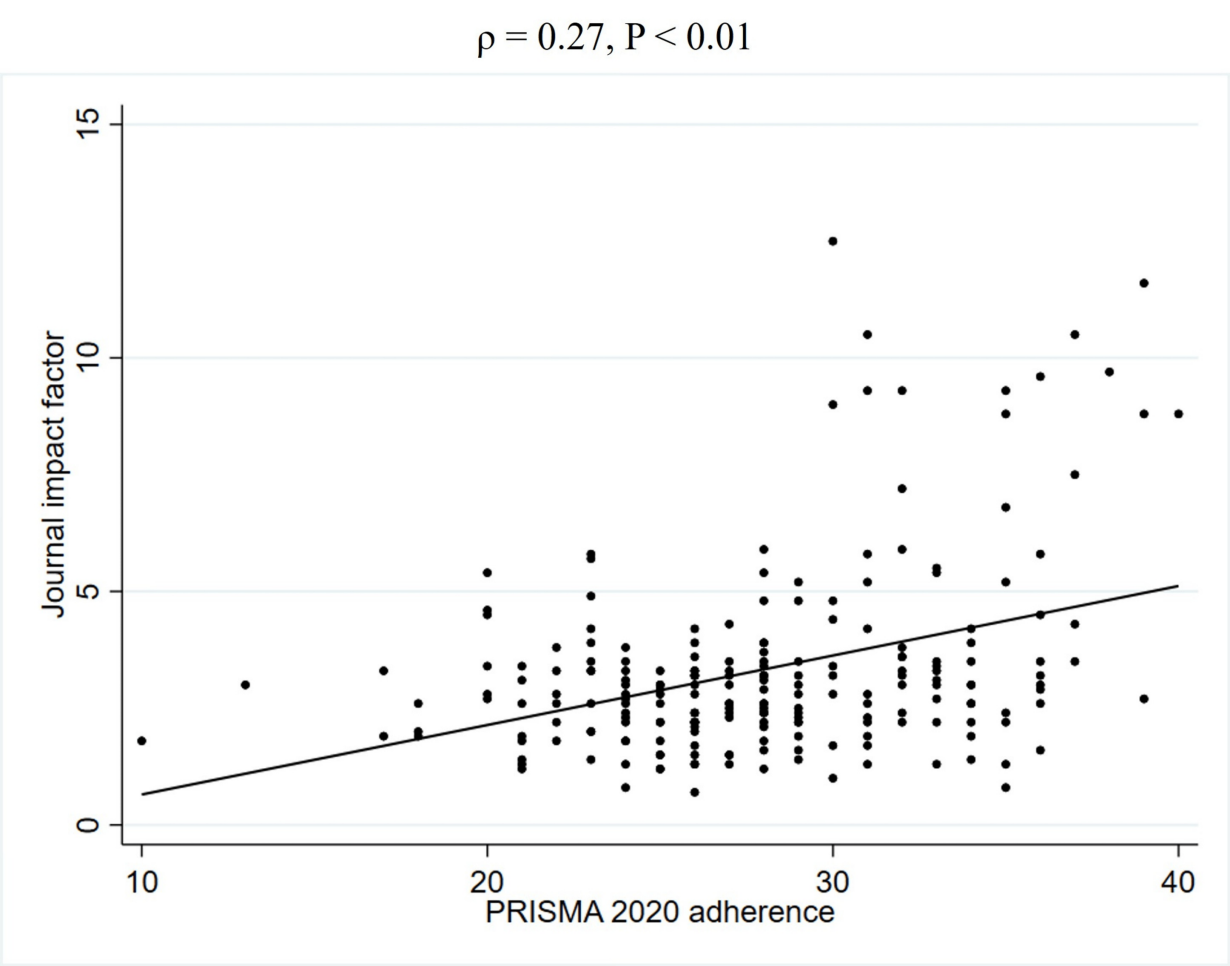
Spearman's rank correlation coefficients between PRISMA 2020 adherence and JIF PRISMA: Preferred Reporting Items for Systematic reviews and Meta-Analyses; JIF: Journal impact factor

**Figure 3:**
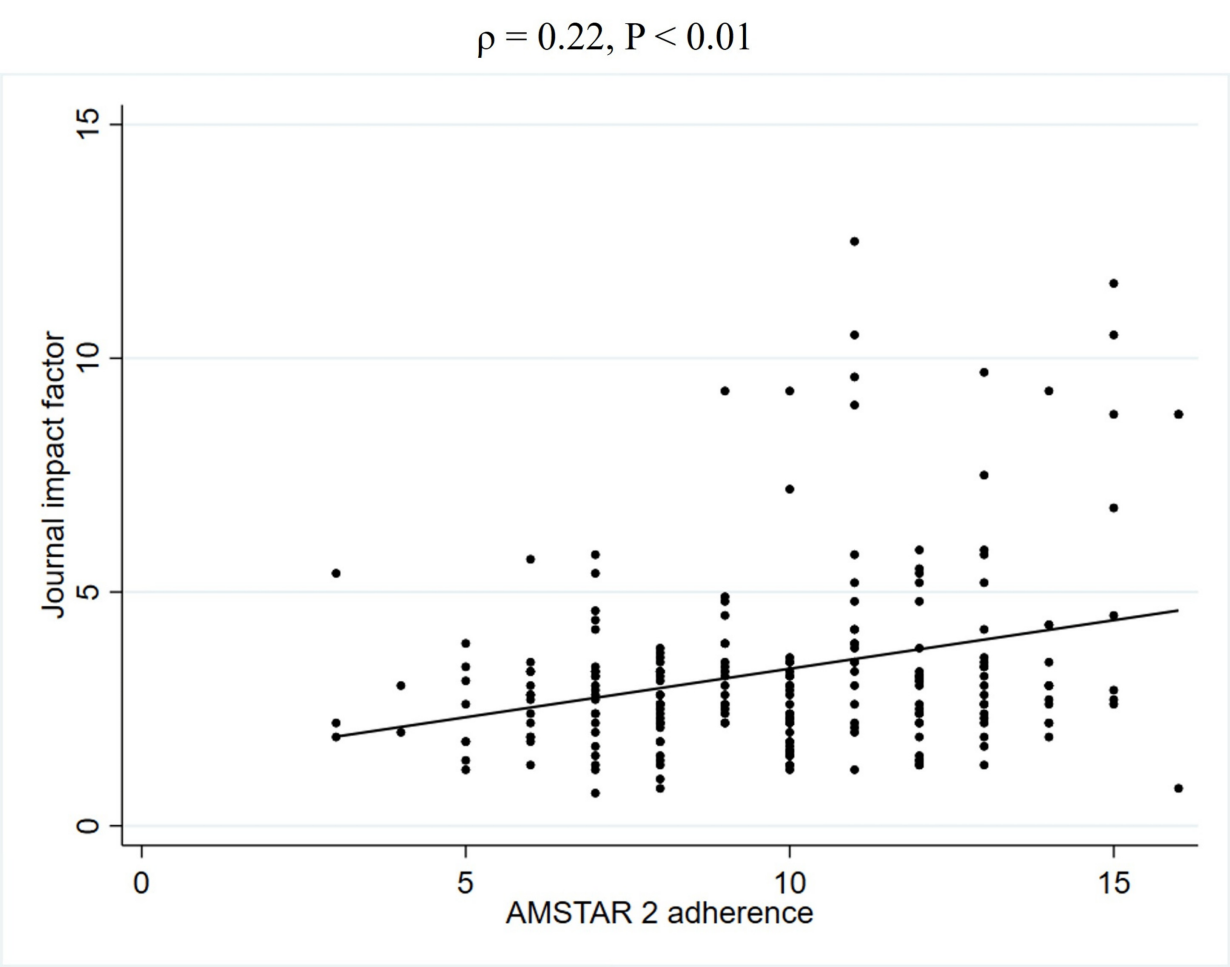
Spearman's rank correlation coefficients between AMSTAR 2 adherence and JIF AMSTAR: A MeaSurement Tool to Assess systematic Reviews; JIF: Journal impact factor

**Figure 4:**
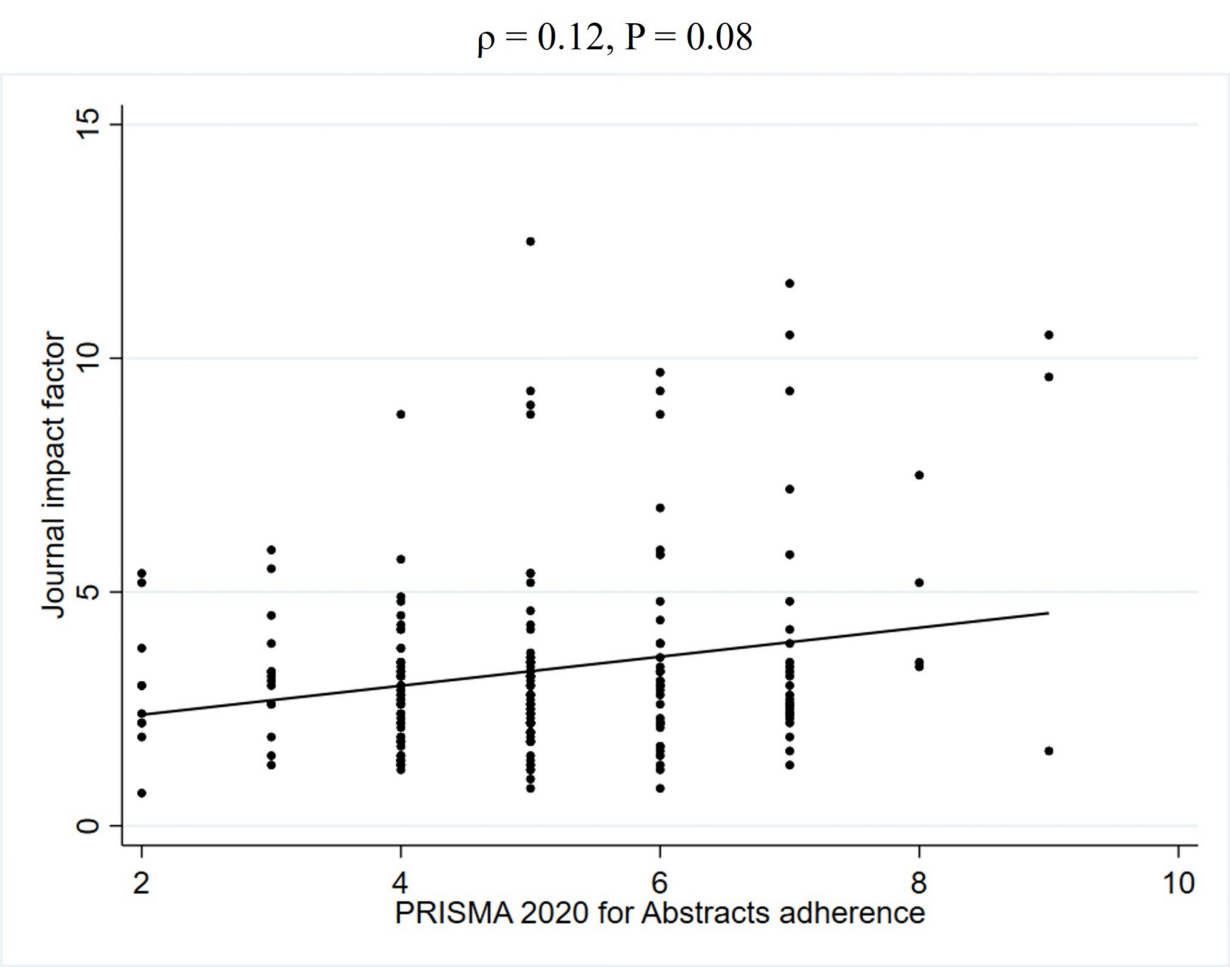
Spearman's rank correlation coefficients between PRISMA 2020 for abstracts adherence and JIF PRISMA: Preferred Reporting Items for Systematic reviews and Meta-Analyses; JIF: Journal impact factor

**Figure 5:**
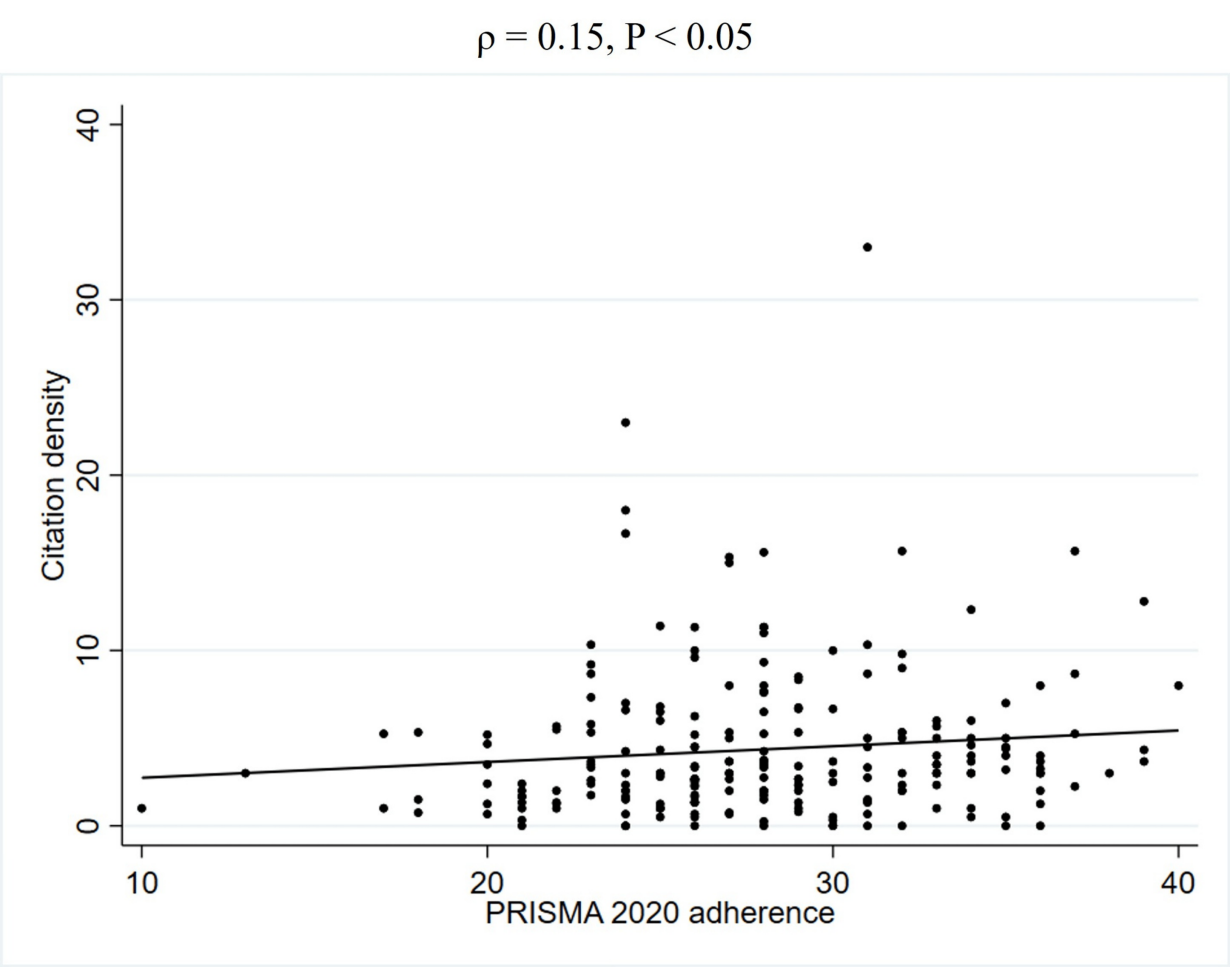
Spearman's rank correlation coefficients between PRISMA 2020 adherence and citation density. PRISMA: Preferred Reporting Items for Systematic reviews and Meta-Analyses

**Figure 6:**
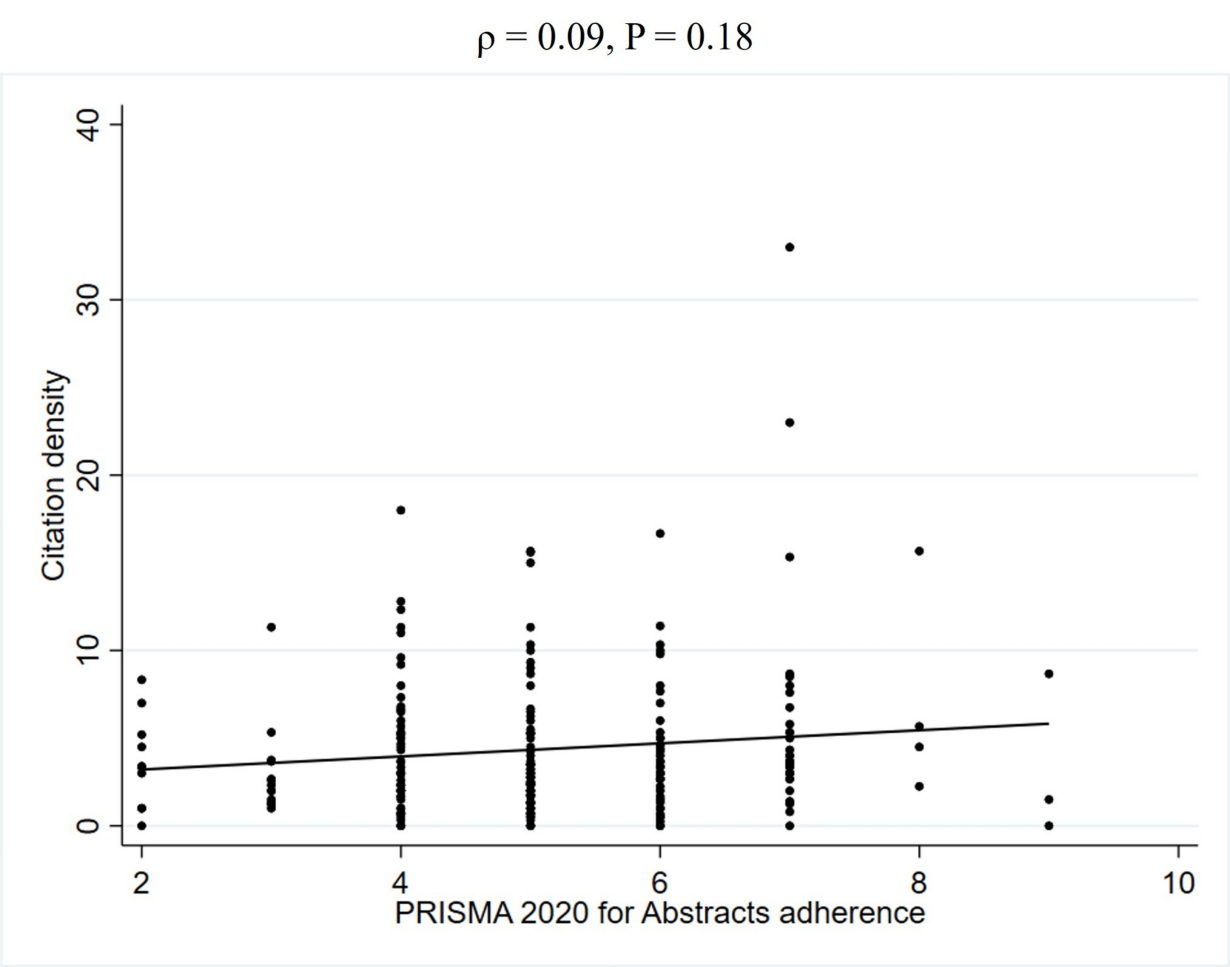
Spearman's rank correlation coefficients between PRISMA 2020 for abstracts adherence and citation density. PRISMA: Preferred Reporting Items for Systematic reviews and Meta-Analyses

**Figure 7:**
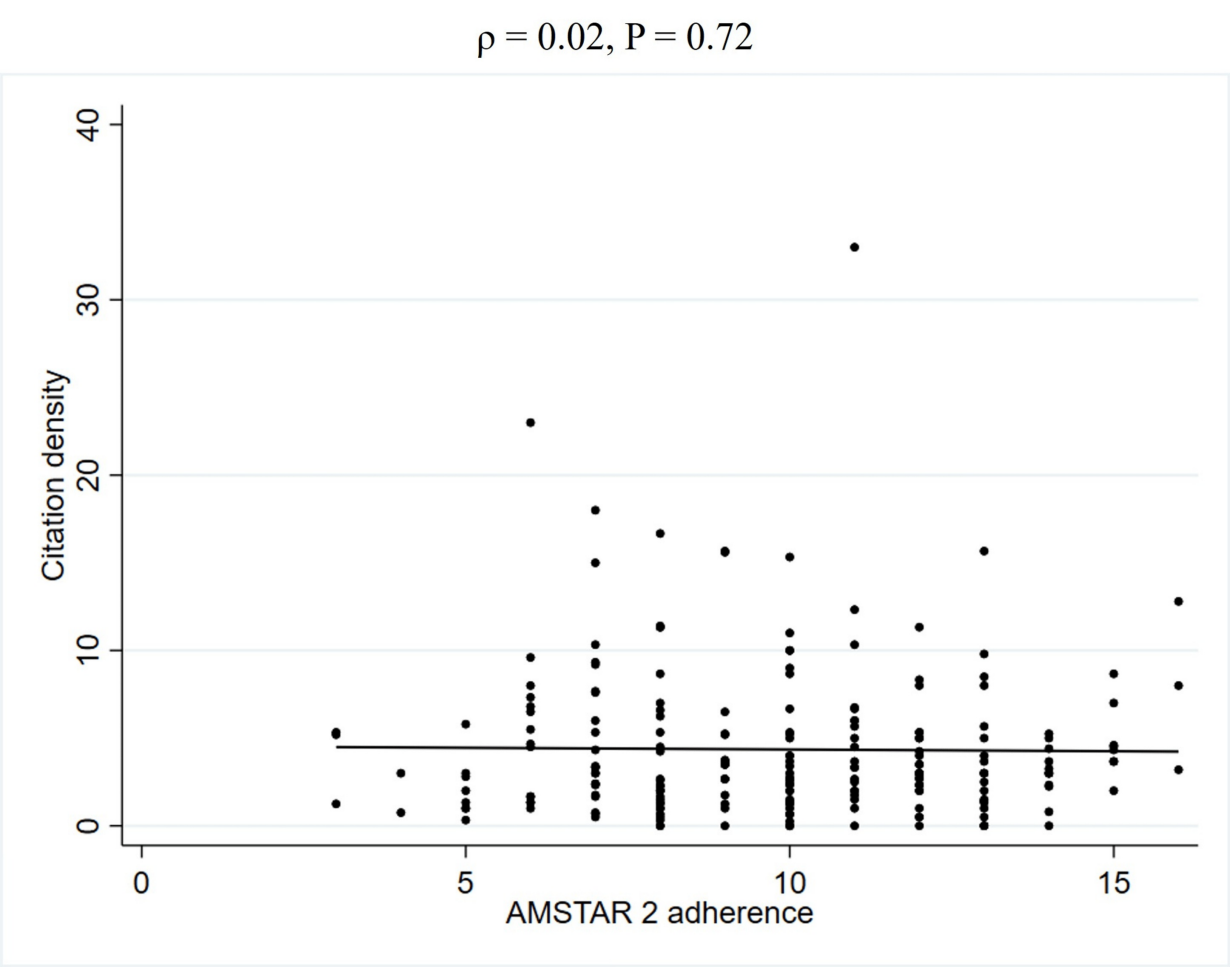
Spearman's rank correlation coefficients between AMSTAR 2 adherence and citation density AMSTAR: A MeaSurement Tool to Assess systematic Reviews

**Figure 8:**
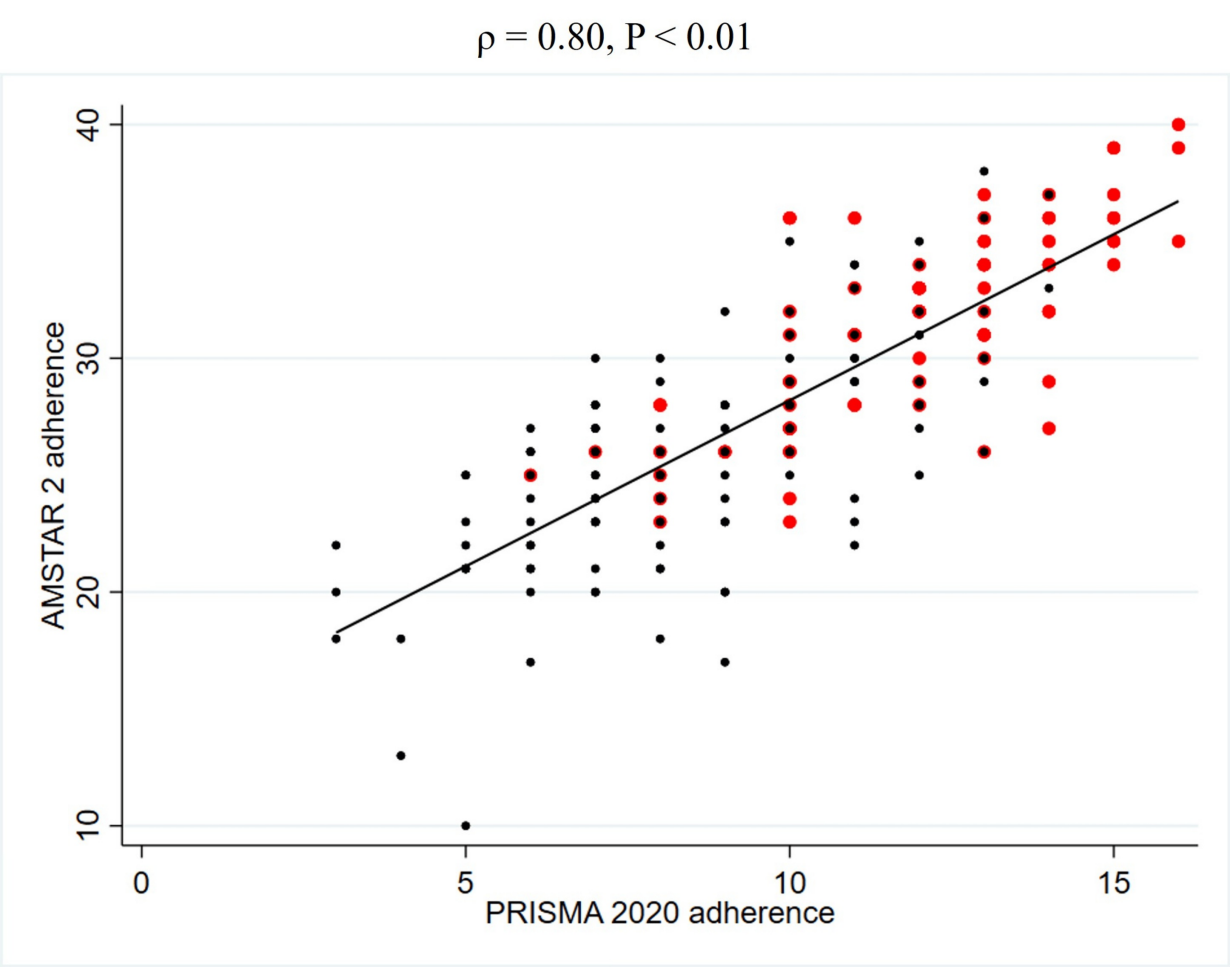
Spearman's rank correlation coefficients between PRISMA 2020 adherence and AMSTAR 2 adherence Red dots represent SRs that used GRADE, and black dots represent SRs that did not use GRADE. PRISMA: Preferred Reporting Items for Systematic reviews and Meta-Analyses; AMSTAR: A MeaSurement Tool to Assess systematic Reviews; SR: Systematic review; GRADE: Grading of Recommendations Assessment, Development and Evaluation

To further explore the unadjusted relationship between quality scores and JIF, Spearman's rank correlation coefficients were calculated (Table 5). This analysis revealed a weak positive correlation between PRISMA 2020 adherence and the JIF (2023) (ρ = 0.27, P < 0.01) (Figure 2, Table 5). Similarly, a weak positive correlation was observed between AMSTAR 2 adherence and the JIF (2023) (ρ = 0.22, P < 0.01) (Figure 3, Table 5). In contrast, no significant correlation was found between PRISMA 2020 for abstracts adherence and the JIF (2023) (ρ = 0.12, P = 0.08) (Figure 4, Table 5). Regarding the correlation between these adherence and citation density, a very weak positive correlation was observed for PRISMA 2020 adherence (ρ = 0.15, P < 0.05), while no significant correlations were found for the others (Figures 5, 6, 7, Table 5). The Spearman's rank correlation coefficient between PRISMA 2020 adherence and AMSTAR 2 adherence revealed a very strong positive correlation (ρ = 0.80, P < 0.05) (Figure 8).

**Table 5:** Spearman's rank correlation coefficients (ρ) of PRISMA 2020, PRISMA for abstracts, and AMSTAR 2 adherence with citation density and JIF (2023) * P < 0.05; ** P < 0.01. PRISMA: Preferred Reporting Items for Systematic reviews and Meta-Analyses; AMSTAR: A MeaSurement Tool to Assess systematic Reviews; JIF: Journal Impact Factor

Variable	PRISMA 2020 adherence (ρ)	PRISMA 2020 for abstracts adherence (ρ)	AMSTAR 2 adherence (ρ)
Citation density	0.15 *	0.09	0.02
JIF (2023)	0.27 **	0.12	0.22 **

Discussion

This meta-epidemiological study investigated factors associated with the quality of SRs in rehabilitation journals. Our primary findings reveal that adherence to key components of PRISMA 2020, specifically use of GRADE and protocol registration, is significantly associated with higher reporting and methodological quality. Furthermore, we observed a weak but statistically significant positive correlation between the JIF and the quality of SRs.

Our findings advocate for researchers to prioritize adherence to PRISMA 2020, as its core components are intricately linked with the quality of SRs in rehabilitation. Our analysis identified that use of GRADE and protocol registration-both key practices promoted by PRISMA 2020-were significantly associated with higher quality. Indeed, PRISMA 2020 incorporates the assessment of the certainty of evidence (CoE), often performed using GRADE, as an essential element of robust reporting [5]. The GRADE system requires assessing risk of bias, publication bias, imprecision, inconsistency, and indirectness, which aligns with key criteria in both PRISMA 2020 and AMSTAR 2 [20-24]. Despite its importance, the application of CoE assessment via the GRADE system remains suboptimal within rehabilitation journals [25]. Therefore, our findings suggest that the broader implementation of the GRADE framework is associated with higher overall quality of SRs in this domain. Similarly, protocol registration is associated with higher quality SRs, as it is intended to enhance transparency and mitigate bias through the pre-specification of review methods [26]. In contrast, our study found no significant association between review quality and the mere reporting of PRISMA 2020 use or journal endorsement of its recommendations. These findings suggest that genuine, item-by-item adherence to PRISMA 2020 checklist is essential, rather than a superficial declaration of its application [7].

In the field of rehabilitation, our findings suggest that higher-quality SRs tend to be published in journals with a relatively high JIF. This observation, based on a weak yet statistically significant positive correlation between JIF and the quality of SRs, suggests that JIF may serve as a potential indicator of review quality in rehabilitation journals. This finding is consistent with prior reports that SRs in higher-JIF core clinical journals tend to have superior methodological quality [11]. Journals with higher JIFs often have more stringent author guidelines requiring adherence to reporting standards and trial registration, which likely contributes to the observed improvements in both reporting and methodological quality [27]. Conversely, our analysis revealed no significant correlation between AMSTAR 2 adherence and citation density. This finding aligns with previous research suggesting that methodological quality does not always translate directly to citation impact, as factors beyond methodological rigor-such as the novelty or scope of a topic-may exert a stronger influence on the citation rate of an SR [28]. These findings on proxy metrics highlight the imperative for strict adherence to PRISMA and GRADE during the review process, alongside researchers' critical appraisal of SR quality before citation. It is also important to recognize that the JIF itself is influenced by a multitude of factors, including editorial processes and open access policies [29]. Therefore, the JIF should be considered just one of several indicators reflecting a journal's influence and, by extension, the potential quality of the SRs it publishes.

Our study has several notable strengths. First, to our knowledge, this is the first study to specifically investigate factors associated with the reporting and methodological quality of SRs published in rehabilitation journals. Second, study selection and data extraction were performed in duplicate by two independent reviewers to minimize potential bias and enhance the reliability of our findings. Third, our use of widely recognized and validated tools - PRISMA 2020 for reporting quality and the AMSTAR 2 for methodological quality - strengthens the validity and comparability of our assessments.

Nevertheless, this study has several limitations. First, our findings may not be fully generalizable, as our analysis was restricted to English-language SRs from a single database and focused on the field of rehabilitation. This potential for selection bias is noteworthy; while most journals with a JIF are indexed in the MEDLINE (PubMed) database, our findings may not extend to journals that lack a JIF and are accessible only through other databases. Second, as a secondary analysis, our study was inherently limited by the variables and assessment methods of the original dataset, although we attempted to mitigate this limitation by extracting additional relevant variables, such as the JIF and citation density. Third, as a meta-epidemiological study with an observational design, our analysis can only identify associations and cannot establish a causal relationship between the identified factors and the quality of systematic reviews.

## Conclusions

Our study revealed that adherence to PRISMA 2020, particularly through practices such as using GRADE and protocol registration, is significantly associated with the reporting and methodological quality of SRs in rehabilitation journals. This finding highlights that the substantive application of these core practices, rather than nominal compliance, is crucial for building a trustworthy evidence base for clinical practice. Therefore, to enhance the reporting and methodological quality of SRs in rehabilitation, researchers should prioritize adherence to PRISMA 2020, including the use of GRADE and protocol registration, which were identified as key factors associated with higher quality.
